# Aortic Remodelling Is Improved by 2,3,5,4′-Tetrahydroxystilbene-2-O-*β*-D-glucoside Involving the Smad3 Pathway in Spontaneously Hypertensive Rats

**DOI:** 10.1155/2015/789027

**Published:** 2015-11-29

**Authors:** Ju Duan, Xin Han, Shuang Ling, Woting Gan, Li Sun, Rong-Zhen Ni, Jin-Wen Xu

**Affiliations:** Murad Research Institute for Modernized Chinese Medicine, Shanghai University of Traditional Chinese Medicine, Shanghai 201203, China

## Abstract

Hypertension is a common health problem that substantially increases the risk of cardiovascular disease. The condition increases blood pressure, which causes alterations in vascular structure and leads to the development of vascular pathologies. 2,3,5,4′-Tetrahydroxystilbene-2-O-*β*-D-glucoside (THSG), a resveratrol analogue extracted from a Chinese medicinal plant, has been proven to have numerous vascular protection functions. This study investigated whether THSG can improve vascular remodeling, which has thus far remained unclear. Orally administering THSG to spontaneously hypertensive rats (SHRs) aged 12 weeks for 14 weeks significantly inhibited intima-media thickness in the lower parts of the aortic arch, increased the vascular diastolic rate in response to acetylcholine, and reduced remodelling-related mRNA expression, such as that of ACTA2, CCL3, COL1A2, COL3A1, TIMP1 WISP2, IGFBP1, ECE1, KLF5, MYL1 BMP4, FN1, and PAI-1. Immunofluorescence staining also showed an inhibitory effect similar to that of THSG on PAI-1 protein expression in rat aortas. Results from immunoprecipitation and a Western blot assay showed that THSG inhibited the acetylation of Smad3. A chromatin immunoprecipitation assay showed that THSG prevented Smad3 binding to the PAI-1 proximal promoter in SHR aortas. In conclusion, our results demonstrated that the inhibitory effect of THSG on aortic remodelling involved the deacetylating role of Smad3 with increasing blood flow and with constant blood pressure.

## 1. Introduction

TGF-*β* is implicated in restenosis and attributed to TGF-*β*-mediated intimal thickening and arterial remodeling [1]. TGF-*β* expression and activation in SHR aortas has been firmly established to be higher than in WKY rats [2, 3]. TGF-*β* proteins are the main regulators of blood vessel development and maintenance. Zacchigna et al. recently reported that TGF-*β* regulation is linked to blood pressure homeostasis [4]. Furthermore, TGF-*β* is a crucial factor for vascular remodeling [5] and abundant collagen matrix deposition is dependent on endogenous TGF-*β* activity [6]. TGF-*β* also induces PAI-1 expression, and Smad3 directly mediates TGF-*β* signalling through direct binding to a TGF-*β* responsive element in the PAI-1 promoter [6, 7].

The resveratrol glucoside THSG, extracted from the medicinal plant* Polygonum multiflorum* Thunb. that is widely used in traditional Chinese medical hospitals, has been demonstrated in various studies to inhibit the proliferation of vascular smooth muscle cells, phosphorylation of Rb, and expression of cyclin D1 induced by low-density lipoprotein and oxidised low-density lipoprotein [8, 9]; attenuate inflammation, including NF*κ*B, and the expression of iNOS in rats' aortic walls or cerebral ischemia injury tissue [10]; reduce arachidonate metabolism and the formation of 5-hydroxy-6,8,11,14-eicosatetraenoic acid, 12-hydroxyheptadecatrienoic acid, and thromboxane B2 [11]; prevent vascular endothelial dysfunction in atherogenic-diet rats [12]; and weaken neointimal hyperplasia in a rat carotid artery balloon injury model [13]. THSG also exhibits other biological activities, such as protecting against staurosporine-induced toxicity in cultured rat hippocampal neurons [14], relieving gastrointestinal motility disorders in STZ-induced diabetic mice [15], and improving diabetic nephropathy [16]. Some studies have concluded that, after oral administration of THSG, the major distribution tissues in rats are in the heart, kidney, livers, and lungs [17], and levels of plasma and tissues of rats were in the range of 0.27–185.00 *μ*g/mL [18]. In cytotoxicity, the published results demonstrated that hepatotoxicity of THSG had not been found in hepatic cells in the range of 400 *μ*mol/L [19] and in rat livers intragastrically injected with 300 mg/kg of THSG for 60 days [20]. Recently, it was reported that THSG can, at least partially, prevent vascular senescence and improve blood flow through the SirT1-eNOS axis, both in vitro and in vivo [21]. In our pharmacological function screening of ingredients, we found that THSG can directly activate the NAD^+^ dependent histone deacetylase activity of SirT1, and based on a report on the pharmacological function of THSG, this study investigated whether THSG can improve vascular remodelling.

## 2. Materials and Methods

### 2.1. Animals

Male SHRs and age-matched WKY negative control rats aged 11 weeks were purchased from the Shanghai Experimental Animal Center of Academia Sinica (Shanghai, China). The animals were handled in compliance with the Guide for the Care and Use of Laboratory Animals, and animal experiments in the study were approved by the Animal Ethics Committee of Shanghai University of Traditional Chinese Medicine. Body weight and rat tail blood pressure were measured before group allocation to ensure homogeneity. Rats were housed 3 per cage at constant humidity (65 ± 5%) and temperature (24 ± 1°C) in a 12-hour light-dark cycle and allowed ad libitum access to tap water and food throughout the experimental protocols. SHRs were adapted to the environment for 1 week and then randomly divided into 2 groups of 8 animals each, the THSG treatment group and the control group. For 14 weeks, 50 mg/kg per day of THSG was administered orally to rats in the experimental groups. Animals in the normal control SHR and negative control WKY groups received equivalent amounts of adjuvant sodium carboxymethyl cellulose. Systolic blood pressure (SBP, tail-cuff method) and weight were measured weekly. The average of 3 measurements was calculated to determine the initial mean SBP. On the last day of treatment, the animals were anaesthetised using an intraperitoneal injection of pentobarbital at a dose of 40 mg/kg of body weight. A small incision was made to the cervical region of each rat to expose the carotid artery, which was cannulated and connected to the blood pressure transducer of an 8-channel PowerLab recorder to measure central arterial blood pressure, blood flow, and heart rate.

### 2.2. Vascular Ring Preparation and Isometric Vascular Tone Recording

Vascular ring preparation and isometric vascular tone recording were performed as described previously [22]. In brief, on the 14th week of THSG administration, thoracic aortas from 4 rats (aged 26 weeks) of each experimental group were removed after anaesthesia and dissected in ice-cold Krebs solution. After connective tissue and fat were removed, the aortas were cut into rings approximately 3 mm wide. All dissection procedures were performed with extreme care to protect the endothelium from damage. Some endothelial layers were mechanically removed by gently rubbing the luminal surface of the aortic ring with a wooden toothpick. Endothelial integrity or functional removal was verified by the presence or absence of the relaxant response to acetylcholine (ACh). With the use of 2 L-shaped stainless-steel wires inserted into the lumen, the aortic rings were suspended in a tissue bath containing Krebs solution (pH 7.4, containing [mmol/L] NaCl 118, KCl 4.7, MgSO_4_ 1.1, KH_2_PO_4_ 1.2, CaCl_2_ 1.5, NaHCO_3_ 25, and glucose 10) at 37°C, while being continuously bubbled with 95% O_2_ and 5% CO_2_. The baseline load placed on the aortic rings was 2 g and the changes in isometric tension were recorded using a force-displacement transducer connected to a computer running PowerLab (ADInstruments Company, Dunedin, New Zealand). After equilibration for 1 hour, the aortic rings were contracted twice using KCl at 60 mmol/L to obtain the maximal response, and the rings were then washed with Krebs solution every 20 minutes until the tension returned to the basal level. The relaxant response curve to ACh (10^−9^–10^−5^ mol/L) was obtained for endothelium-intact and endothelium-deprived aortic rings contracted with 10^−6^ mol/L phenylephrine (PE) and then treated with NO donor sodium nitroprusside at 5 *μ*mol/L to induce complete relaxation through vasodilation. The aortic rings were washed 3 times with fresh Krebs solution and then either vasorelaxation or vasoconstriction was examined.

### 2.3. Histological Study

To evaluate aortic medial wall thickness, haematoxylin and eosin stained aortic sections were photographed. Descending aortic arch intima-media thickness (IMT) was measured from the inner border of the lumen to the outer border of the tunica media. The average of 3 sectional measures was calculated for each animal.

### 2.4. Immunoprecipitation and Western Blot

The cleared aortas and cultured vascular smooth muscle cells were lysed in ice-cold RIPA buffer (50 mmol/L Tris/HCl, pH 8.0, 150 mmol/L NaCl, 2 mmol/L sodium orthovanadate, 1% Nonidet-P40, 1% sodium deoxycholate, 0.1% SDS, 0.1 mmol/L DTT, 0.05 mmol/L PMSF, 0.002 mg/mL of aprotinin, and 0.002 mg/mL of leupeptin). Lysates were precleared through centrifugation at 12 000 g for 10 minutes at 4°C. For immunoprecipitation, anti-Smad3 [AF9F7] antibody (ab75512, 5 *µ*g/mL) was added to the precleared lysates for 1 hour at 4°C and incubation continued overnight at 4°C with protein A/G plus agarose (50 *µ*L/mL). Immunoprecipitates were washed 5 times with 1 mL of ice-cold PBS. Immunoprecipitated proteins were eluted from the agarose beads by boiling for 5 minutes in SDS sample buffer. Aliquots of the cell lysate (50 or 100 *µ*g of each sample) were resolved on SDS-PAGE and transferred to nitrocellulose membranes. The membranes were blocked in 5% skim milk overnight at 4°C. This was followed by incubation with primary (anti-Smad3 or anti-acetyl-lysine) antibodies for 2 hours and exposure to a horseradish peroxidase-conjugated secondary antibody at room temperature for 1 hour. Visualisation was performed using an ECL Immobilon Western Chemiluminescent horseradish peroxidase (HRP) substrate (Millipore, Billerica, MA, USA). Quantitative analysis of band density was performed using ImageJ software from NIH. Western blot experiments were performed in triplicate.

### 2.5. Immunofluorescence Staining

Frozen sections (6 *μ*m thick) were cut from a cryostat and transferred onto gelatin-coated glass slides. The sections were fixed in sucrose-cacodylate buffer (0.1 mol/L sodium cacodylate, 0.1 mol/L sucrose, and 0.25% glutaraldehyde) for 15 minutes and permeabilised with 0.5% Triton X-100 in PBS. The sections were blocked with 1% bovine serum albumin in PBS at room temperature for 30 minutes. To detect the expression of plasminogen activator inhibitor-1 (PAI-1), the sections were incubated with primary antibodies against PAI-1 (ab66705) (1 : 500, Abcam, Cambridge, UK) followed by goat polyclonal antirabbit FITC-labelled (ab6717) secondary IgG antibodies (1 : 1000, Abcam). In partial staining, 4′,6-diamidino-2-phenylindole (DAPI) was used as a histological background control. Immunofluorescence imaging was performed manually with a 40x objective lens (camera: DP70; ISO: 200; Tv: 10 seconds for PAI-1) of an Olympus IX71 fluorescence microscope (Olympus, Tokyo, Japan).

### 2.6. RNA Isolation and Quantitative PCR

Total RNA was extracted from the rat aortas with the Invitrogen TRIzol reagent (Life Technologies, Grand Island, USA) according to the manufacturer's instructions. First-strand cDNA was synthesised using a High-Capacity cDNA Reverse Transcription Kit (Life Technologies). The cDNA was mixed with Maxima SYBR Green qPCR Master Mix (Fermentas, Burlington, Canada) and gene-specific primers (Table 1) (Shanghai Generay Biotech, Shanghai, China). The sequences of the primers are shown in Table 1. Quantitative polymerase chain reaction (PCR) was performed using 7500 Fast Real-Time PCR Systems (Applied Biosystems, Foster City, USA) according to the manufacturer's instructions. The amplification conditions comprised an initial 15-minute denaturation step at 95°C, followed by 40 cycles of denaturation at 95°C for 15 seconds, annealing at 55°C for 30 seconds, and elongation at 72°C for 30 seconds. The dissociation curves were analysed to ensure the amplification of a single PCR product. Three independent assays were performed for each primer. The amount of cDNA was calculated for each sample from the standard curve. The relative expression is shown after normalisation by GAPDH gene expression.

### 2.7. Chromatin Immunoprecipitation Assay

A ChIP assay was performed using an assay kit (Abcam, Cambridge, UK) according to the manufacturer's instructions. The sliced aortas were cross-linked with 1% formaldehyde at room temperature for 10 minutes. Glycine was added to stop the cross-linking. After being washed with ice-cold PBS, the aortas were lysed for 10 minutes with the addition of 200 *µ*L of SDS lysis buffer in 20 mg of aortic tissue fragments. The chromatin was sheared through sonication 4 times for 40 seconds at one-third maximum power, with 1 minute of cooling on ice between each pulse. Cross-linked chromatin was quantitated to determine the initial amount of DNA present in the various samples (input chromatin). The remaining chromatin fractions were precleared with protein A-agarose for 1 hour and immunoprecipitated with the antibodies of interest (10 *µ*L) overnight at 4°C. Immune complexes were collected with protein A-agarose for 1 hour, washed 5 times, and eluted in 1% SDS, 0.1 M NaHCO_3_. Cross-linking was reversed overnight at 65°C and samples were digested with 100 *µ*g of proteinase K at 45°C for 1 hour. DNA was then recovered in DEPC-H_2_O for PCR. PCR was performed using rat PAI-1 proximal region-specific primers (Table 2). PCR products were analysed through electrophoresis on 3% agarose gels. The optical density of each PCR fragment was estimated (GIS 2009 UV Transilluminator, Tanon Science & Technology Co., Ltd. Shanghai, China) and sample-specific PCR fragment density was used to calculate the equivalence point.

### 2.8. Statistical Analysis

The results are presented as means ± SEM. For parametric data, comparisons among treatment groups were performed with an ANOVA test of variance using SPSS (SPSS Software, Inc, Northampton, MA, USA) and post hoc tests for each figure. Values of *P* < 0.05 were considered statistically significant.

## 3. Results

### 3.1. THSG Improved Aortic Remodelling and Vascular Diastolic Rate In Vivo

**Figure 1:**
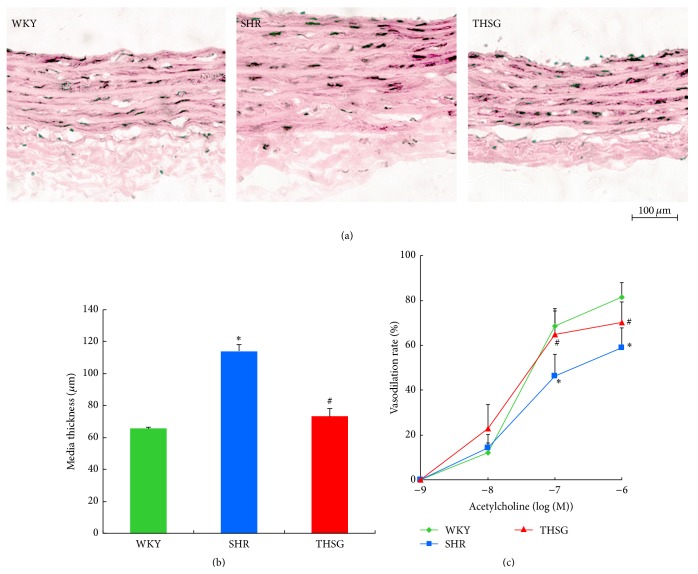
THSG improved vascular wall thickening in rat aortas. (a) Haematoxylin and eosin (H&E) staining for aortal sections from WKY rats, SHRs, and SHRs treated with THSG. (b) The media thickness of aortas from WKY rats, SHRs, and SHRs treated with THSG (*n* = 5). (c) ACh induced an endothelium-dependent relaxation at the indicated dose in PE precontracted rat aortic rings (*n* = 4). The maximum vasodilation by sodium nitroprusside (50 *μ*mol/L) was considered as 100%. Data are expressed as the mean ± SEM. ^*∗*^ *P* < 0.05 versus WKY; ^#^ *P* < 0.05 versus SHRs.

Previous studies have shown that THSG can inhibit vascular senescence and improve blood flow but does not change blood pressure [21]. Additional experiments were conducted to clarify the effect of THSG on vascular remodelling. As shown in Table 3 and Supplemental Figure  1A (see Supplementary Material available online at http://dx.doi.org/10.1155/2015/789027), THSG failed to reduce mean central arterial and rat tail blood pressure and did not affect the weight of the heart, liver and kidney, or body but did improve blood flow. As illustrated in Figure 1(a), IMT, number of the elastic layers, and spacing between the elastic layers in the lower parts of the aortic arch were notably increased in aortas from SHRs compared with aortas from WKY rats. By contrast, administering THSG to rats aged 12 weeks for 14 weeks significantly inhibited aortic IMT in SHRs. Aortic IMT in SHRs was higher than that in WKY rats, increasing from 131.2 ± 4.8 to 211.8 ± 6.6 *μ*m (*n* = 4, ^*∗*^ *P* < 0.05, SHR versus WKY) (Figures 1(a) and 1(b)). THSG almost completely suppressed thickening; aortic IMT in rats given THSG was reduced to 165.6 ± 10.4 *μ*m (*n* = 4, ^#^ *P* < 0.05, THSG versus SHR) (Figures 1(a) and 1(b)). The number of the elastic layers and the spacing between the elastic layers are also greatly reduced in SHR aortas treated with THSG compared with nontreated SHR group (Figure 1(a)). The vascular ring of aorta from administration groups have been shown to improve the vasodilation response to acetylcholine. On stimulation with 0.1 *μ*mol/L and 1 *μ*mol/L acetylcholine, the aortic diastolic rate of WKY rats reached 68.7 ± 7.8% and 81.5 ± 6.5%, respectively, in response to 10^−7^ or 10^−6^ *μ*mol/L acetylcholine. By contrast, the SHR aortic diastolic rate was only 46.2 ± 9.8% and 59.0 ± 8.7% (*n* = 3, ^*∗*^ *P* < 0.05, SHR versus WKY) (Figure 1(c)). In groups given THSG, the aortic diastolic rate was 64.9 ± 10.4% and 70.3 ± 9.1% (*n* = 3, ^#^ *P* < 0.05, THSG versus SHR) (Figure 1(c)). These results suggest that THSG can effectively inhibit aortic remodelling.

### 3.2. THSG Inhibited Aortic-Remodelling-Related Gene Expression

**Figure 2:**
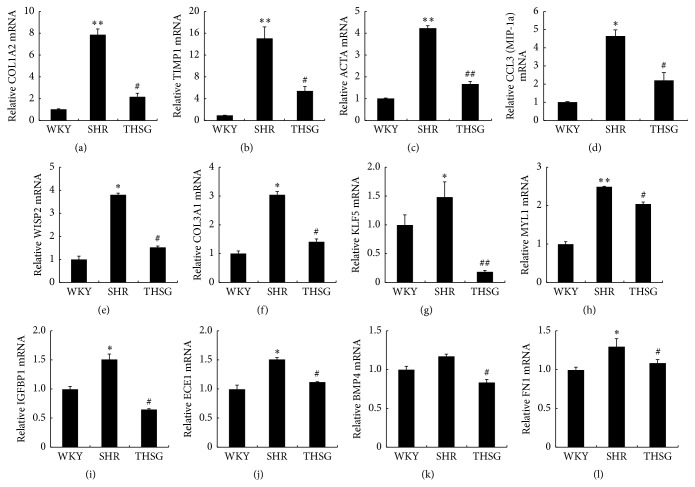
THSG inhibited vascular-remodelling-related gene expression. Total RNA was extracted from rat aortas by using Invitrogen TRIzol reagent. ((a) to (l)) COL1A2, ACTCA, TIMP1, CCL3, IGFBP1, ECE1, KLF5, MYL1, WISP2, COL3A1, BMP4, and FN1 mRNA expression was measured using a Maxima SYBR Green qPCR Master Mix kit. Data are normalised to GAPDH mRNA expression levels (*n* = 4). Data are expressed as the mean ± SEM. ^*∗*^ *P* < 0.05 versus WKY, ^*∗∗*^ *P* < 0.01 versus WKY, ^#^ *P* < 0.05 versus SHRs; ^##^ *P* < 0.01 versus SHRs.

**Figure 3:**
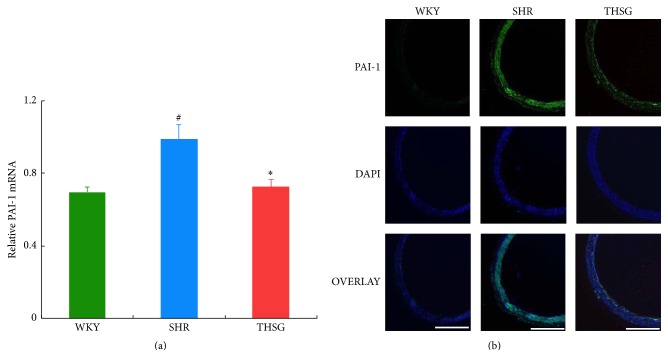
Influence of THSG on PAI-1 expression. (a) Rat PAI-1 mRNA detected using quantitative RT-PCR in aortas of WKY rats, SHRs, and SHRs treated with THSG (*n* = 3). Densitometric analysis was performed based on 3 separate experiments. Data are expressed as the mean ± SEM. ^#^ *P* < 0.01 versus WKY; ^*∗*^ *P* < 0.01 versus SHRs. (b) Aortal sections were incubated with primary antibodies against PAI-1, followed by FITC-labelled rabbit IgG secondary antibodies. DAPI fluorescence staining was used as control. Immunofluorescence staining was performed using a fluorescence microscope. Scale bar = 500 *μ*m.

To determine the effect of THSG on the expression of remodelling-related genes in aortas, the mRNA levels of ACTA2, CCL3, COL1A2, TIMP1, IGFBP1, ECE1, KLF5, MYL1, WISP2, COL3A1, BMP4, FN1, and PAI-1 (Serpin E1) were examined using quantitative real-time PCR. The results showed that expression of ACTA2, CCL3, COL1A2, COL3A1, TIMP1, WISP2, IGFBP1, ECE1, KLF5, MYL1 BMP4, and FN1 in SHR aortas increased by severalfold (each *n* = 3, ^#^ *P* < 0.05; ^##^ *P* < 0.01 versus WKY) (Figure 2). However, administering THSG significantly reduced the expression of ACTA2, CCL3, COL1A2, COL3A1, TIMP1 WISP2, IGFBP1, ECE1, KLF5, MYL1 BMP4, and FN1 genes from SHR aortas to the level of these genes in WKY aortas (each *n* = 3, ^*∗*^ *P* < 0.05; ^*∗∗*^ *P* < 0.01 versus SHRs) (Figure 2). As shown in Figure 3(a), SHRs had higher PAI-1 mRNA expression in aortas than WKY rats had (*n* = 3, *P* < 0.05). Administering THSG significantly suppressed PAI-1 expression in SHR aortas (each *n* = 3, *P* < 0.05). In addition, immunofluorescence staining showed a similar tendency of PAI-1 protein expression (Figure 3(b)).

### 3.3. THSG Inhibited Smad3 Acetylation and DNA Binding Ability In Vivo

**Figure 4:**
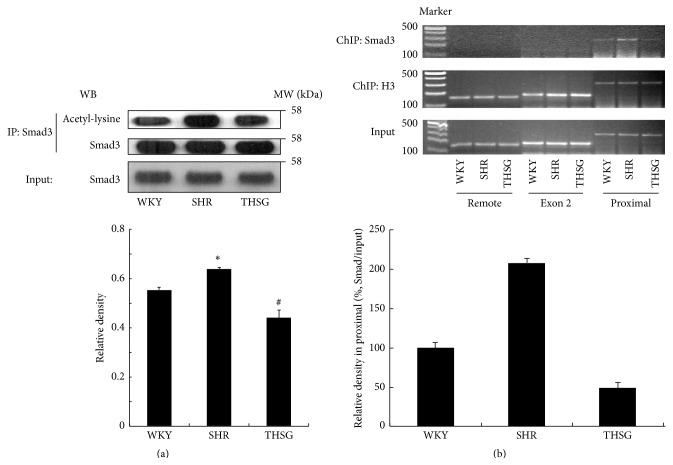
Effect of THSG on Smad3 deacetylation and DNA binding ability in vivo. (a) Acetylation of Smad3 detected using immunoprecipitation and Western blot assays in aortas from WKY rats, SHRs, and SHRs treated with THSG (*n* = 3). Data are expressed as the mean ± SEM. ^*∗*^ *P* < 0.01 versus WKY; ^#^ *P* < 0.01 versus SHRs. (b) Smad3 binding activation was determined using a chromatin immunoprecipitation assay on promoter proximal, remote, and exon 2 regions of rat PAI-1 gene in aortas from WKY rats, SHRs, and SHRs treated with THSG. Bottom panel: Smad3 binding relative densities in rat PAI-1 proximal promoter were analyzed (*n* = 2).

Early studies showed that, in TGF-*β*1-stimulated cells [3], Smad-binding elements (SBEs) on −280, −580, and −730 base pair regions can bind to Smad3 in the PAI-1 promoter [23]. The expression of TGF-*β* in the primary aortic smooth muscle cells in SHR aortas is higher than that in WKY rats [24], suggesting that TGF-*β*1 signalling at least partially controls PAI-1 expression in SHRs. Therefore, the role of the acetylation stat of Smad3 in the regulation of PAI-1 expression was examined. Using immunoprecipitation and Western blot assays, it was discovered that Smad3 in SHRs was more acetylated (Figure 4(a)). However, administering THSG repressed acetylation of Smad3 in SHR aortas (*n* = 3, *P* < 0.05, Figure 4(a)). A chromatin immunoprecipitation assay was performed to evaluate the binding capability of Smad3 in the PAI-1 promoter from rat aortas. The results confirmed that Smad3 binds proximal regions of the PAI-1 promoter (Figure 4(b), upper panel). In SHR aortas, the binding capability of Smad3 was distinctly higher than that in WKY, whereas binding was reduced in rats treated with THSG (*n* = 2, Figure 4(b)).

## 4. Discussion

### 4.1. Role of PAI-1 in Vascular Remodelling

PAI-1 is a fast-acting inhibitor of plasminogen activation. Previous studies have highlighted that the pharmacological inhibition and genetic deficiency of PAI-1 attenuate angiotensin II/salt-induced aortic remodeling [25]. Elevated PAI-1 in hypertension is related to metabolic risk factors for cardiovascular disease [26]. Aortas of SHRs exhibited higher levels of PAI-1 expression [27], which is associated with vascular remodelling and increased vascular wall component stiffness [28]. Our study revealed that the expression of vascular-remodelling-related genes, such as ACTA2, CCL3, COL1A2, COL3A1, TIMP1 WISP2, IGFBP1, ECE1, KLF5, MYL1 BMP4, FN1, and PAI-1, was elevated in SHR aortas, a finding that is consistent with previous reports. Patients with essential hypertension exhibit varying degrees of IMT increases in carotid arteries [29]. Some studies have shown that differences in carotid IMT are associated positively with PAI-1 expression [26]. This study determined that aortic IMT was notably increased in SHRs, suggesting that aortas in SHRs exhibit significant vascular remodelling.

### 4.2. Beneficial Effect of THSG on Cardiovascular Remodelling

THSG can inhibit the proliferation of vascular smooth muscle cells [9, 30], improve vascular endothelial function [11], and increase eNOS gene expression [31] and balloon-injury-induced neointimal hyperplasia [12]. Our previous studies showed that THSG can prevent vascular senescence [21]. This study demonstrated that THSG can suppress the increase of aortic IMT and reduce vascular-remodelling-related gene expression, such as that of ACTA2, CCL3, COL1A2, COL3A1, TIMP1 WISP2, IGFBP1, ECE1, KLF5, MYL1 BMP4, FN1, and PAI-1, in SHR aortas, suggesting that THSG can improve vascular remodelling. On the basis of the THSG being an analogue of resveratrol, a similar study reported that resveratrol cannot reduce the blood pressure of SHR rats over 20 weeks old and that resveratrol partially attenuates the vascular remodelling process and might be related to the ability of resveratrol to alleviate oxidative stress in SHR and enhance protein kinase G activity [32]. The ability of resveratrol to prevent vascular remodeling is better than its inhibitory effect on rise in blood pressure, on which THSG effect is similar. One study reported that resveratrol can lower blood pressure [33]. However, the 3-4-week-old SHR rats in that study had an early hypertensive status, in which the pathologic condition differs from that of rats to more than 20 weeks old, which may not yet have formed vascular remodelling. In addition, some studies have shown that resveratrol inhibits SHR vascular smooth muscle cell growth and collagen synthesis [34] and enhances endothelium-dependent relaxation [35], showing similarity with our results.

### 4.3. Smad3 and PAI-1

Some studies have demonstrated that p300 interacts with Smad2 and Smad3 in a ligand-dependent manner and enhances the transactivation by Smads [36] and that Smad3 transcriptional activity is regulated by acetylation at Lys-378 by p300/CBP [37]. Similarly, acetylation of Lys-19 enhanced the DNA binding activity of Smad3 [38, 39]. This study demonstrated that Smad3 was more acetylated in aortas of SHR compared with that of WKY rats and that administration of THSG repressed Smad3 acetylation in SHR aortas, which is supported by our previous studies showing that THSG activated SirT1 activity and expression [21]. We also verified that Smad3 can bind to the proximal regions of the PAI-1 promoter in SHR aortas, which contain 2 response elements of TGF-*β* signalling. By contrast, this binding was reduced in rats treated with THSG. These findings reveal that THSG obstructs vascular remodelling and PAI-1 expression through SIRT1-mediated deacetylation of Smad3.

### 4.4. Significant Improvement of Vascular Remodelling with Increasing Blood Flow and with Constant Blood Pressure

As shown in Table 3 and Supplemental Figure 1A, our results showed that THSG improved vascular remodelling with increasing blood flow and with constant blood pressure, similar to the effect of resveratrol [31]. Appropriate increasing blood flow is beneficial for vasodilation and vasoprotection, because blood-flow-related shear stress is a major stimulus of NO release from the endothelium [40, 41]. Recently, studies have also shown that some inhibitors improve vascular remodelling without affecting blood pressure, despite some contradictory research results. The Halmosi group [42, 43] reported that treatment with L-2286, a PARP inhibitor, exerts a protective effect in hypertension-induced vascular and myocardial remodelling through modulating the expression and activity of heat shock proteins, Akt-1/GSK-3*β*, and several PKC isoforms and does not affect the blood pressure measured using the tail-cuff method. Another study reported that the PPAR*γ* activator rosiglitazone prevents l-NAME-induced inward hypertrophic remodeling of cerebral arteries without affecting blood pressure [44]. Dipeptidylpeptidase-4 (DPP-4) inhibitor linagliptin can also ameliorate cardiovascular injury in salt-sensitive hypertensive rats independently of blood glucose and blood pressure [45]. In addition, imatinib mesylate, a specific tyrosine kinase inhibitor, also prevents diastolic dysfunction and attenuates myocardial remodelling in SHRs and significantly reduces the mRNA expression of collagen I, collagen III, and PDGF receptor-*β* phosphorylation but does not affect blood pressure [46]. The inhibitory effects of pharmaceutical ingredients, including THSG and resveratrol, on remodelling rather than blood pressure suggest that all improvement process of low-grade sterile chronic inflammation, oxidative stress, apoptosis, and fibrosis may delay vascular remodelling. For example, THSG can attenuate inflammation, including NF*κ*B [9], reduce arachidonate metabolism and the formation of thromboxane B2 [10], and inhibit neointimal hyperplasia in a rat carotid artery [13]. Therefore, additional long-term experiments may be able to reduce arterial blood pressure accompanied by improvement in vascular function, but hypertension cause, such as the renin-angiotensin system disorders, has not been removed. In summary, it is clear that THSG improves blood flow and vascular remodelling without affecting blood pressure, and perhaps this may be important for improving vascular function.

In conclusion, administering THSG reduced ACTA2, CCL3, COL1A2, COL3A1, TIMP1 WISP2, IGFBP1, ECE1, KLF5, MYL1 BMP4, FN1, and PAI-1 (Serpin E1) expression in aortas and improved the aortic IMT of SHRs. THSG inhibited acetylation of Smad3 in vivo and in vitro and attenuated binding of Smad3 to the proximal regions of the PAI-1 promoter. Collectively, our results demonstrated the inhibitory effect of THSG on aortic remodelling, involving the Smad3 pathway. In the future, we need to clarify whether THSG can reverse the existing IMT in older rats.

## Supplementary Material

Supplementary Figure 1. Effect of THSG on rat tail blood pressure (A) and body weight (B) from 13 weeks to 25 weeks of age in SHRs and WKY rats (each n = 8).

## Figures and Tables

**Table 1:** qRT-PCR primers.

Gene	Primer	Size (bp)	GenBank number
ACTA2 (a-SMA)	Forward 5′-TGACCCAGATTATGTTTGAGACCTT-3′	110	NM_031004.2
	Reverse 5′-AGAGTCCAGCACAATACCAGTT-3′		

CCL3 (MIP-1a)	Forward 5′-CTGCTGCTTCTCCTATGG-3′	111	NM_013025.2
	Reverse 5′-CGGTTTCTCTTGGTCAGG-3′		

COL1A2	Forward 5′-TCGCTCACAGCCTTCACTCAG-3′	119	NM_053356.1
	Reverse 5′-TGCGGGCAGGGTTCTTTCTA-3′		

TIMP1	Forward 5′-CATCCTCTTGTTGCTATCATTG-3′	159	NM_053819.1
	Reverse 5′-ATCTCATAACGCTGGTATAAGG-3′		

PAI-1 (Serpin E1)	Forward 5′-AAGAGCCAGATTCATCATC-3′	198	NM_012620.1
	Reverse 5′-GTGCTACCATCAGACTTG-3′		

CTGF	Forward 5′-ATGCTGTGAGGAGTGGGTGTGT-3′	202	NM_022266.2
	Reverse 5′-GGCAGTTGGCTCGCATCATAGT-3′		

Gapdh	Forward 5′-ACAGCAACAGGGTGGTGGAC-3′	252	NM_017008.3
	Reverse 5′-TTTGAGGGTGCAGCGAACTT-3′		

**Table 2:** Chromatin immunoprecipitation primers.

PAI-1 gene	Location	Primer
Promoter proximal	−312~−636	Forward 5′-GGGAGTCAGAGGTGCTTCAC-3′
		Reverse 5′-GCCTACAGTGCCTGGTGTTT-3′

Promoter remote	−4223~−4381	Forward 5′-GGAACTGTCCCAGAAATCCA-3′
		Reverse 5′-TCAGCTTCTGACCCTTTGGT-3′

Exon 2 region	+2772~+2950	Forward 5′-CTCTCCACCCAGTCGTTGAT-3′
		Reverse 5′-ATGGGGTCATGGAACAAGAA-3′

**Table 3:** The parameters for each experimental animal group.

	WKY	SHR	THSG
Number	8	8	8
Age (weeks)	26	26	26
Body weight (g)	428.6 ± 7.8	437.1 ± 6.5	421.2 ± 8.1
Heart weight (g)	1.3 ± 0.0	1.6 ± 0.1	1.5 ± 0.0
Liver weight (g)	10.3 ± 0.9	12.0 ± 0.2	12.3 ± 0.6
Kidney weight (g)	1.3 ± 0.0	1.4 ± 0.0	1.4 ± 0.0
Heart rate (beats/min)	352.7 ± 26.1	376.4 ± 21.3	325.5 ± 24.1
Mean arterial pressure (mmHg)	179.3 ± 12.6	235.7 ± 8.15^*∗*^	231.9 ± 16.0
Blood flow (mL/min)	4.7 ± 0.5	4.5 ± 0.4	6.4 ± 0.6^#^

^*∗*^ *P* < 0.05 versus WKY; ^#^ *P* < 0.05 versus SHR.

## Abbreviations

ACTA2: Actin, alpha 2, smooth muscle, aorta
BMP4: Bone morphogenetic protein 4
CCL3: Chemokine (C-C motif) ligand 3
ChIP: Chromatin immunoprecipitation assay
COL1A2: Collagen, type I, alpha 2
COL3A1: Collagen, type III, alpha 1
DAPI: 4′,6-Diamidino-2-phenylindole
ECE1: Endothelin converting enzyme 1
FN1: Fibronectin 1
GAPDH: Glyceraldehyde-3-phosphate dehydrogenase
HRP: Horseradish peroxidase
IGFBP1: Insulin-like growth factor binding protein 1
IMT: Intima-media thickness
KLF5: KLF5 Kruppel-like factor 5
MYL1: Myosin, light chain 1, alkali, skeletal, fast
PAI-1: Plasminogen activator inhibitor 1
qPCR: Real-time quantitative PCR detecting
TGF-*β*: Transforming growth factor-*β*
TIMP1: Tissue inhibitor of metalloproteinases 1
SHR: Spontaneously hypertensive rat
THSG: 2,3,5,4′-Tetrahydroxystilbene-2-O-*β*-D-glucoside
WISP2: WNT1-inducible-signaling pathway protein 2
WKY rat: Wistar Kyoto rat.
