# Supplementary material for: Aortic Remodelling Is Improved by 2,3,5,4′-Tetrahydroxystilbene-2-O-β-D-glucoside Involving the Smad3 Pathway in Spontaneously Hypertensive Rats
Source: Evid Based Complement Alternat Med. 2015 Nov 29;2015:789027. doi: 10.1155/2015/789027 (PMC4677031; doi:10.1155/2015/789027)

**Supplemental Materials**

**Supplemental Figure Legends**

**Supplemental-Figure I**

Effect of THSG on rat tail blood pressure (A) and body weight (B) from 13 weeks to 25 weeks of age in SHRs and WKY rats (each n = 8).

**Supplemental Figures**

Supplemental-Figure I


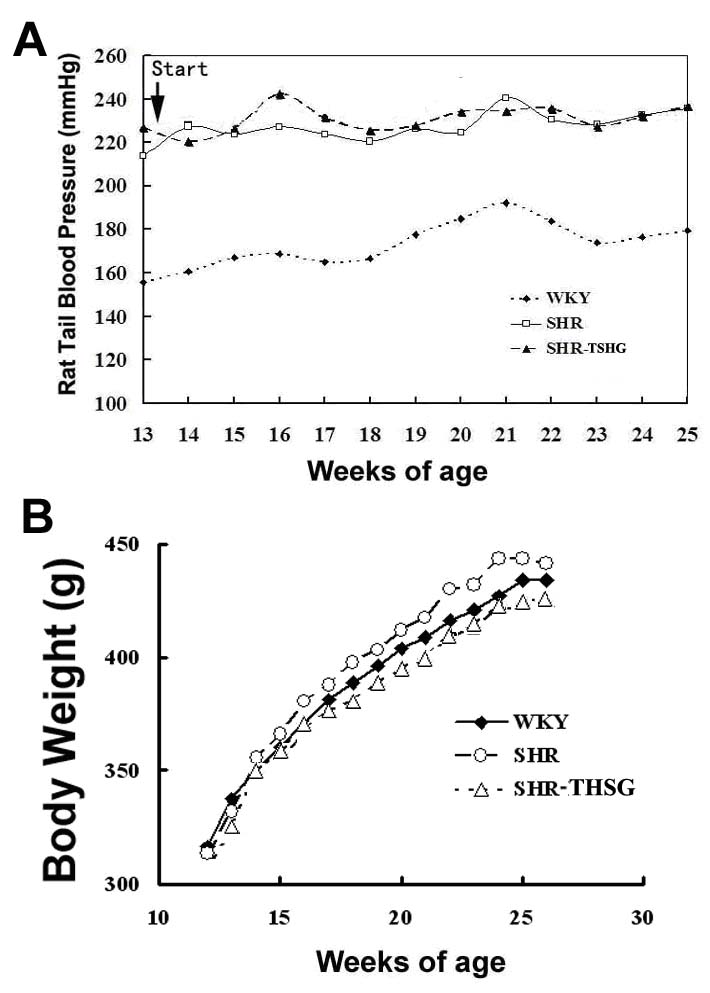

Supplement: Supplementary file 1 — Supplementary Figure 1. Effect of THSG on rat tail blood pressure (A) and body weight (B) from 13 weeks to 25 weeks of age in SHRs and WKY rats (each n = 8). [file 789027.f1.docx]
